# Systematic changes in position sense accompany normal aging across adulthood

**DOI:** 10.1186/1743-0003-11-43

**Published:** 2014-03-25

**Authors:** Troy M Herter, Stephen H Scott, Sean P Dukelow

**Affiliations:** 1 Hotchkiss Brain Institute, University of Calgary, Calgary, Alberta, Canada; 2 Department of Clinical Neurosciences, University of Calgary, Calgary, Alberta, Canada; 3 Centre for Neuroscience Studies, Queen’s University, Kingston, Ontario, Canada; 4 Department of Anatomy and Cell Biology, Queen’s University, Kingston, Ontario, Canada; 5 School of Medicine, Queen’s University, Kingston, Ontario, Canada; 6 Deprtment of Exercise Science, University of South Carolina, Columbia, South Carolina, USA

**Keywords:** Proprioception, Position sense, Upper limb, Robotics, Assessment, Aging

## Abstract

**Background:**

Development of clinical neurological assessments aimed at separating normal from abnormal capabilities requires a comprehensive understanding of how basic neurological functions change (or do not change) with increasing age across adulthood. In the case of proprioception, the research literature has failed to conclusively determine whether or not position sense in the upper limb deteriorates in elderly individuals. The present study was conducted a) to quantify whether upper limb position sense deteriorates with increasing age, and b) to generate a set of normative data that can be used for future comparisons with clinical populations.

**Methods:**

We examined position sense in 209 healthy males and females between the ages of 18 and 90 using a robotic arm position-matching task that is both objective and reliable. In this task, the robot moved an arm to one of nine positions and subjects attempted to mirror-match that position with the opposite limb. Measures of position sense were recorded by the robotic apparatus in hand-and joint-based coordinates, and linear regressions were used to quantify age-related changes and percentile boundaries of normal behaviour. For clinical comparisons, we also examined influences of sex (male versus female) and test-hand (dominant versus non-dominant) on all measures of position sense.

**Results:**

Analyses of hand-based parameters identified several measures of position sense (Variability, Shift, Spatial Contraction, Absolute Error) with significant effects of age, sex, and test-hand. Joint-based parameters at the shoulder (Absolute Error) and elbow (Variability, Shift, Absolute Error) also exhibited significant effects of age and test-hand.

**Conclusions:**

The present study provides strong evidence that several measures of upper extremity position sense exhibit declines with age. Furthermore, this data provides a basis for quantifying when changes in position sense are related to normal aging or alternatively, pathology.

## Background

Proprioception refers to the ability to perceive the location of one’s body in space and has been classically divided into two subcomponents: position sense and kinesthesia [1]. Position sense is the ability of an individual to identify the static location of a body part, whereas kinesthesia is the ability to identify body motion. Although muscle spindle afferents are considered to provide the dominant source of information for position sense, cutaneous afferents are also an important source of information, particularly for the more distal joints [2-6].

In healthy individuals, the suggestion that position sense may decline with age across adulthood is not entirely surprising because a number of physiological changes occur in the proprioceptive system with increasing age. Studies have shown that muscle spindles show lower sensitivity [7-9], intrafusal fibres decline in number [10,11], spindle diameters decrease in size [12] and capsular thickness increases [11]. Cutaneous mechanoreceptors also decrease in number with age [13,14]. Each of these natural changes may contribute to declines in position sense.

In addition to peripheral changes that occur with increasing age, modifications to the central nervous system may also contribute to age-related declines in proprioception. For example, decreased grey matter in the post-central gyrus [15,16] and reduced activity in proprioceptive regions of the basal ganglia [17] may contribute to declines in position sense across adulthood. Position sense is also influenced in a task-dependent manner by attention and possibly other cognitive factors [18], thus age-related declines in attention and cognition may contribute to declines in proprioception with increasing age.

In spite of these changes in the peripheral and central aspects of somatosensory processing, there remains disagreement whether position sense in the upper limb actually declines with age (for a review see Goble et al. 2009 [19]). In the upper limb, several small studies and one large study have found that position sense is slightly worse in subjects in their seventh or eighth decade of life as compared to performance in their third [20-23]. However, these studies contradict the findings of a large study by Kokmen et al. [24] that found no effects of age on the sense of joint motion (kinesthesia) at the metacarpophalangeal (MCP) joint. The discrepancy between these studies may reflect differences between position sense and kinesthesia. However, both functions are susceptible to peripheral and central influences of aging, thus the effects of aging on position sense and kinesthesia remain unclear.

A clear understanding of the effects of increasing age on position sense is important clinically for identifying how neurological disorders impact proprioception. Notably, stroke and traumatic brain injury often lead to deficits in position sense [25,26]. Poor position sense also correlates with poor functional outcomes following stroke [27], whereas individuals with intact position sense following stroke have significantly better motor recovery [28-32]. By characterizing the effects of aging on position sense, it is possible to differentiate deficits that are caused by stroke or TBI from those reflecting normal declines due to aging.

We conducted the present study to, a) provide a better understanding of age-related declines in upper limb position sense and b) develop a normative data set that could be used for comparison to clinical populations. To achieve these goals, we have used an objective and reliable assessment of position sense based on robotic technology to quantify arm position sense in a large cohort of 209 healthy individuals between 18 and 90 years of age. To examine age-related declines in upper limb position sense, regression models were developed to quantify the influence of age on measures of position sense. Because neurological disorders, such as stroke, can affect both sexes and both sides of the body, we also examined influences of sex (male versus female) and test-hand (dominant versus non-dominant) to control for these factors in the normative data sets. We considered the potential influence of sex given that age-related changes are often thought to be more prominent in the male brain [15,33-35]. We examined differences between using the dominant and non-dominant arms to probe position sense because of their distinct contributions to various aspects of proprioceptive function [36-41].

## Methods

### Participants

Male and female participants between the ages of 18 and 90 were recruited from the communities of Kingston, Ontario and Calgary, Alberta, Canada. Contact was made through posted flyers, advertisements in local newspapers, and direct communication with families of stroke inpatients at St. Mary’s of the Lake Hospital (Kingston, Ontario) and Foothills Medical Centre (Calgary, Alberta). Participants were excluded from the study if they: 1) had any history of neurological impairments, 2) had any ongoing musculoskeletal problems of the shoulder and/or elbow, and 3) were unable to understand the instructions for the testing procedure. To ensure eligibility for the study, all participants completed a clinical examination, which included a detailed medical history and physical examinations of strength of the upper extremity using the MRC grading system [42], range of motion, and motor control using the Purdue Pegboard [43]. Subjects who did not obtain a normal score on any of these tests were excluded from the study. We also excluded any subject who was unable to understand the instructions for any of the clinical assessments or the robotic assessment. A physician or physical therapist with expertise in stroke rehabilitation performed all clinical examinations. Subjects also completed the 10 item modified version of Edinburgh Handedness Inventory (writing, drawing, throwing, scissors, toothbrush, knife, spoon, broom, match, and lid) to determine their hand dominance [44]. Subjects were considered right-handed, left-handed, and ambidextrous if they obtained scores of 50 to 100, -100 to -50, or -49 to 49, respectively [45]. Before entering the study, all participants provided informed consent. Data from some participants (n = 65) had been collected as control data for a previous study of position sense following stroke [26]. All methods for data collection used in the previous study were identical to those used in the current study. Ethics approval was obtained from the ethics boards of Queen’s University, Providence Care, and the University of Calgary.

### Robotic apparatus

**Figure 1:**
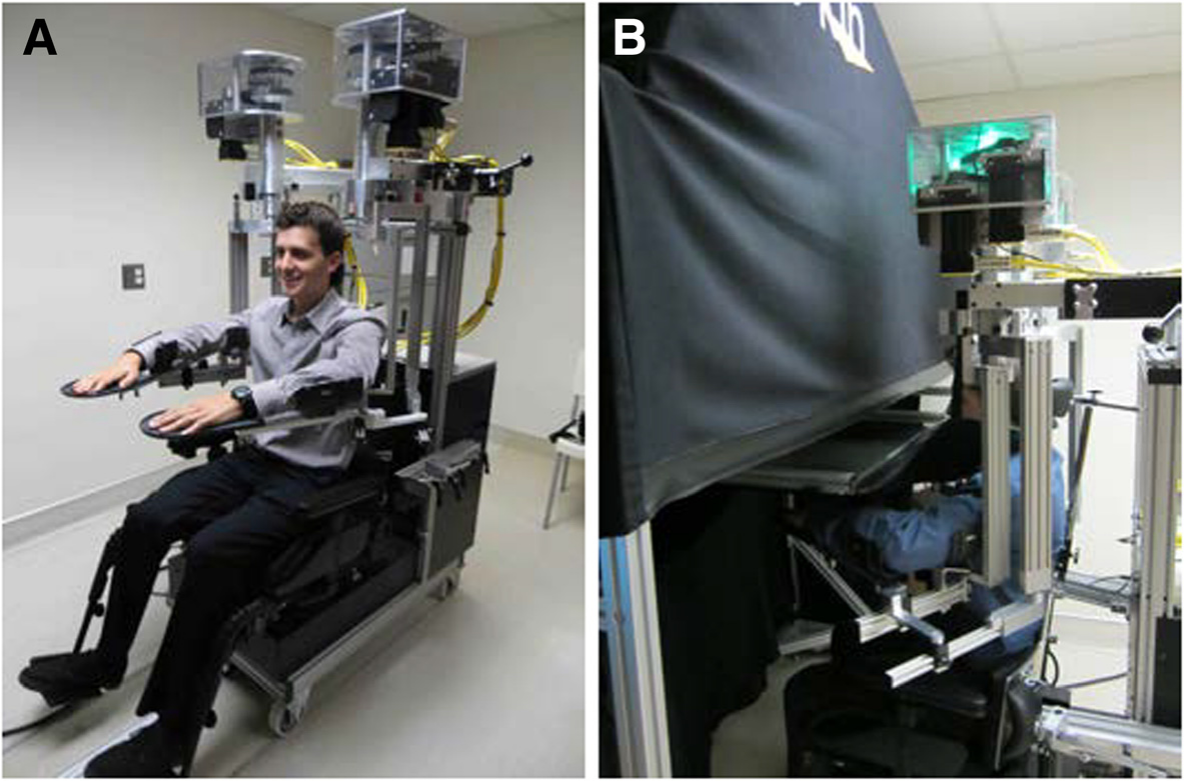
**Robotic apparatus. A**, Frontal view of a participant sitting in the robotic apparatus. The participant sits comfortably in the modified wheelchair base with his arms and hands supported by troughs. **B**, Side view of a participant with the robotic apparatus docked at the augmented reality workstation. Shields mounted under the glass and a soft, black cover hanging between the glass and the participant’s neck occlude all vision of the arms and hands. Written, informed consent for the publication of pictures was obtained from the participant.

The current study used the KINARM exoskeleton robot (BKIN Technologies Ltd., Kingston, Ontario), which is a joint-based robotic apparatus that can be used to manipulate, monitor and record joint- (shoulder and elbow) and hand-based kinematic data (Figure 1). The robotic apparatus has been described in a number of previous publications [25-27,46-48]. Participants sit in a modified wheelchair seat with both arms comfortably placed in arm troughs that are adjusted to the dimensions of each individual (Figure 1A). The exoskeleton provides full gravitational support of the arms and hands, permits arm movements in the horizontal plane, and can apply mechanical loads to the shoulder and/or elbow. Angles of the shoulder and elbow are obtained directly from encoders within the robotic motors, and a calibration process is used to compute joint- and hand-based kinematics, including the position of each index finger within the horizontal plane. The robot is docked to an augmented reality system that can display targets within the same plane as the arms and hands. For purposes of the current study, vision of the arms and hands was occluded and the augmented reality display was not employed (Figure 1B).

Although other studies have used instruments such as dynamometers [49], motion-capture systems [50], inclinometers [51], and light exoskeleton systems [52] to measure position sense, our robotic device offers a number of advantages. Notably, our robotic apparatus allows us to rapidly set-up and calibrate subjects, passively move the arms through smooth trajectories, and couple movements at multiple joints (shoulder and elbow) in joint- or hand- coordinates. In addition, unloaded tasks, such as the arm position matching task, require negligible amounts of strength because the arms are fully supported against gravity. Perhaps most important, however, we are able to objectively obtain valid, reliable, and sensitive measures of sensory, motor, and cognitive function with a single platform. Benefits of using robotic technology for assessment of sensory and motor deficits have also been described in a recent review [53].

### Arm position matching task

**Figure 2:**
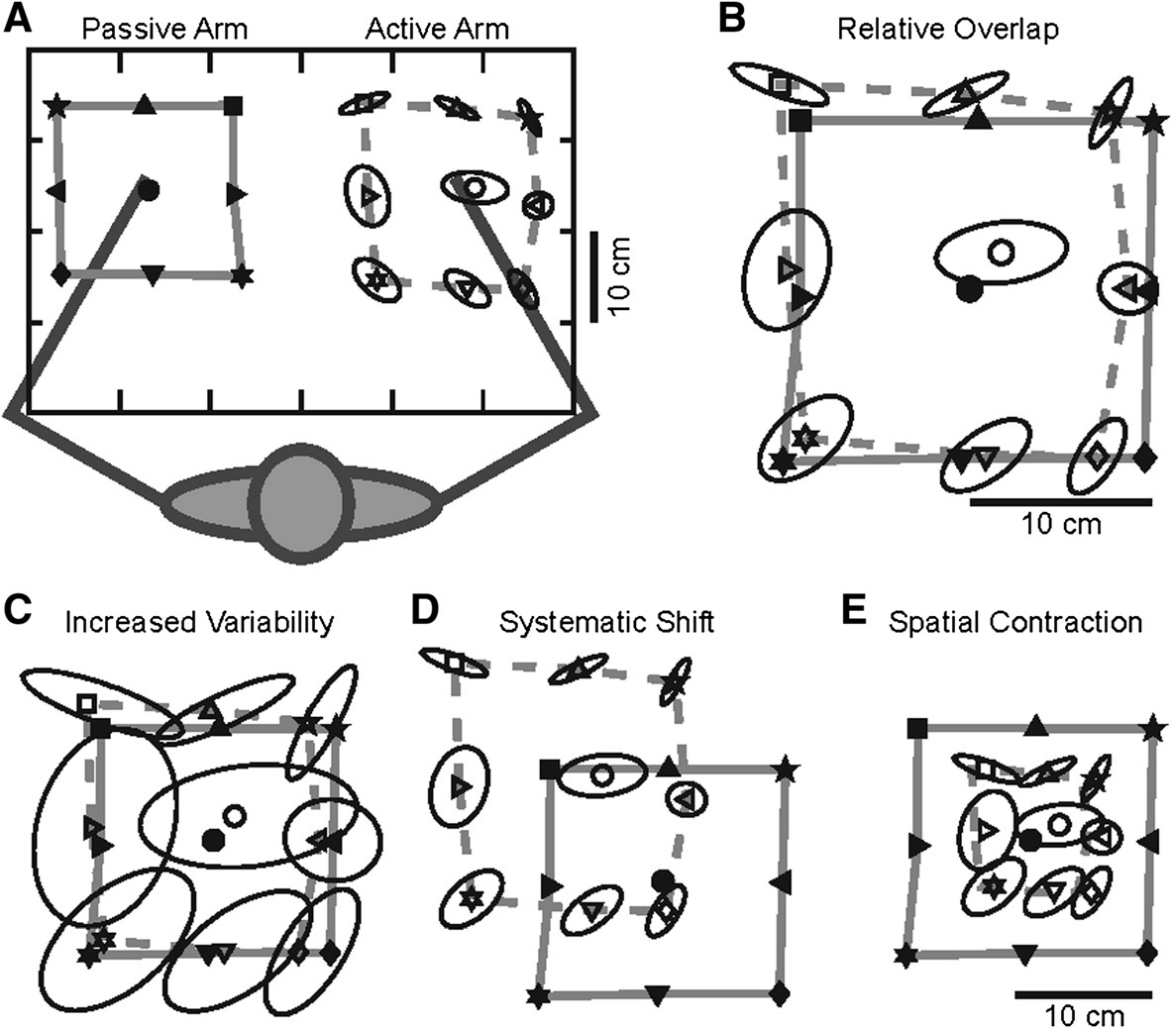
**Position matching task. A**, Top down workspace view of a typical participant. The robot passively moved the left hand to nine spatial locations (filled symbols) and the right arm was actively moved by the participant to mirror-match each spatial location (open symbols). Ellipses around each of open symbols represent one standard deviation. Areas enclosed by the solid and dashed grey lines show the matching areas of passive and active arms, respectively. **B**, Overlap of the passive and active arms after mirror transforming data from the left arm to the right side of the workspace. **C**–**E**, The data has been modified to illustrate examples of increased variability **(C)**, systematic shift **(D)** and spatial contraction **(E)**.

Position sense of the upper extremity was assessed with an arm position matching task [25-27]. With the arms and hands occluded from vision, the robot passively moved one hand (passive hand) to one of nine different target locations (fingertip positions) organized in a 3×3 matrix with 10 cm separation between targets (Figure 2A). The participants were instructed to actively move the opposite hand (active hand) to the mirror location in space. To help ensure that matching performance reflected sensory perception rather than motor control, participants were given as long as needed to complete each trial; the examiner then triggered the next trial when the subjects verbally instructed to the examiner that they felt their active hand was mirror matching their passive hand. By requiring a verbal response before triggering the next trial, we were also able to control for momentary lapses of attention. Each of the nine target locations was presented once in a randomized block. Six different blocks were obtained for a total of 54 trials. All participants completed the task twice, once using each hand as the active hand.

### Analyses

All analyses were performed using MATLAB (Mathworks Inc., Massachusetts, USA). Matching performance was examined in both hand- and joint-coordinates to permit comparisons with both research studies and clinical practice, which commonly use single-joint or whole-limb tasks to measure position sense. Parameters in hand-coordinates were quantified using Cartesian (*x*, *y*) positions of the index fingertips obtained from the robotic apparatus at the end of each trial. Figure 2A illustrates a workspace view of the mean fingertip positions of the passive and active hands of an exemplar participant for each target. In order to visually compare the positions of the passive and active hands, the position of the passive hand was mirrored across the *x* coordinate (Figure 2B).

In a previous study [26], we developed three parameters (Variability (Var), Systematic Shift (Shift), Spatial Contraction/Expansion (C/E) to characterize subject performance based on hand position (See Table 1 for definitions). These parameters showed good to excellent reliability (Var: r = 0.81; Shift: r = 0.70; C/E: r = 0.86). The formulae used to compute Var, Shift, and C/E have been previously described in detail [26]. Separate values for Var and Shift were obtained for the *x*, *y*, and *xy* (linear distance) dimensions. For didactic purposes, Figures 2C, D and E highlight representative patterns of errors, illustrating large variability, a large systematic shift in hand position across the workspace, and a reduction in the overall spatial area of the workspace used with the active hand, respectively. Other studies have generally quantified the absolute errors in position sense [54,55]. These absolute errors should increase due to any of the patterns depicted in Figure 2C-E. For comparison with previous studies, we also computed absolute error (AE).

**Table 1:** Attributes and parameters of the arm position matching task

**Coordinate frame**	**Parameter**	**Abrv**	**Units**	**Definition**
Hand-coordinates	Variability	*Var*	cm	Mean trial-by-trial variability of the active hand in *x*, *y*, and *xy* coordinates.
	Systematic shift	*Shift*	cm	Systematic errors between the mean *x* and *y* positions of the active and passive hands.
	Absolute error	*AE*	cm	Absolute errors between the mean *x* and *y* positions of the active and passive hands.
	Spatial Cont/Exp	*C/E*	—	Ratio of: i) mean spatial area enclosed by the active hand to ii) mean spatial area of enclosed by the passive hand.
Joint-coordinates	Variability	*Var*	deg	Mean trial-by-trial variability of the shoulder and elbow angles of the active arm.
	Systematic shift	*Shift*	deg	Systematic errors between the shoulder and elbow angles of the active and passive arms.
	Absolute error	*AE*	deg	Absolute errors between the shoulder and elbow angles of the active and passive arms.

Parameters in joint-coordinates were quantified using shoulder and elbow angles obtained directly from the robotic apparatus at the end of each trial (See Table 1). In joint-space, separate values were obtained for the shoulder and elbow joints. Each parameter was calculated separately for each target and then averaged across all targets.

Linear regression was used to quantify age-related changes for each parameter. If the grouped data exhibited a significant regression fit (*F*-test, *P* < 0.05), residual values were computed by subtracting out the regression model from the original values. If the grouped data did not exhibit a significant regression fit (*F*-tests, *P* ≥ 0.05), we continued with the original data values.

For those parameters with a significant regression fit, we tested whether the residual values were normally distributed. If the residual values were not normally distributed (Lilliefors test, *P* < 0.05), the original data was transformed with a: 1) logarithmic, 2) square root, or 3) inverse transform. In cases when a transform was needed, linear regression was repeated on the transformed data and, if a significant fit was found (*F*-test, *P* < 0.05), residuals were re-calculated from the transformed data. The residual values were then retested for normality until a normal distribution was obtained from one of the transforms.

After a normal distribution of residual values was obtained, Kolmogorov-Smirnov tests were used to quantify effects of sex (males versus females) and test-hand (dominant versus non-dominant). Note that our examination of test-hand assessed whether subjects performed differently when matching with the dominant hand (left or right) as the active hand compared to using their non-dominant hand as the active hand. Specifically, this did not examine whether right-dominant subjects differ from left-dominant subjects. If a parameter exhibited a significant effect of sex and/or test-hand (*P* < 0.05), the original data was separated by sex (males and females) and/or test-hand (dominant and non-dominant) and the methods described above were repeated to quantify effects of age for each separate group. Data from the separate groups were then used to compute normative statistics.

In order to establish normative statistics for each parameter, we obtained a number of percentiles (1, 2.5, 5, 25, 50, 75, 95, 97.5, 99) from the residual values with a significant effect of age (*F*-test, *P* < 0.05) and the original values without a significant effect of age (*F*-test, *P* ≥ 0.05). Percentiles were obtained using the Matlab percentile function (prctile.m), which uses rank ordering with linear interpolation to find percentiles. Percentiles obtained from a parameter with a significant regression fit could be transformed back into its native units to obtain a unique statistical distribution for any given age, sex, and test-hand. The formulae used for the different inverse transforms include:

(1) $$\text{No}\;\text{Transform}:\;y=\left(\text{age}*\text{slope}\right)+\mathrm{bias}+\text{percentile}$$

(2) $$\text{Log}\;\text{Transform}:\;y=e^{\left(\left(\mathrm{age}*\mathrm{slope}\right)+\mathrm{bias}+\mathrm{percentile}\right)}$$

(3) $$\text{Square}\;\text{Root}\;\text{Transform}:\;y={\left(\left(\text{age}*\text{slope}\right)+\mathrm{bias}+\text{percentile}\right)}^{2}$$

(4) $$\text{Inverse}\;\text{Transform}:\;y=\frac{1}{\left(\left(\text{age}*\text{slope}\right)+\text{bias}+\text{percentile}\right)}$$

Percent changes in parameters were computed using the regression fits to find the median values of parameters for 18 and 90 year-old subjects, then calculating the percent change:

(5) $$\text{Percent}\;\text{Change}:\;\mathrm{\Delta y}=\frac{\left(y_{90}–y_{18}\right)}{y_{18}}*100\%$$

## Results

A total of 209 participants (96 male, 113 female) completed the arm position matching task with both arms. Demographic data describing the age, sex, and handedness of the participants are provided in Table 2. Scores from the Modified Edinburgh Handedness Inventory were used to classify participants as right-handed (handedness score ≥ 50; n = 188), left-handed (handedness score ≤ -50; n = 12), or mixed handedness (-50 < handedness score < 50; n = 9) [44,45]. All nine participants who scored in the mixed handedness range performed more tasks with their right-hand and were, therefore, treated as right-handed participants in our analysis.

**Table 2:** Subject demographics (n= 209 subjects, 96 male and 113 female)

**Age**	**# Subjects**	**Median age**	**Sex**		**Handedness**		
			**Male**	**Female**	**Right (M/F)**	**Left (M/F)**	**Mixed (M/F)**
18 – 29	41	24	16	25	14/22	1/1	1/2
30 – 39	37	34	18	19	17/18	1/1	0/0
40 – 49	34	46	12	22	10/17	1/4	1/1
50 – 59	30	55	8	22	6/21	0/1	2/0
60 – 69	35	63	20	15	18/15	0/0	2/0
70 – 79	23	72	16	7	15/6	1/1	0/0
80 – 90	9	82	6	3	6/3	0/0	0/0

### Hand-based parameter analysis

**Figure 3:**
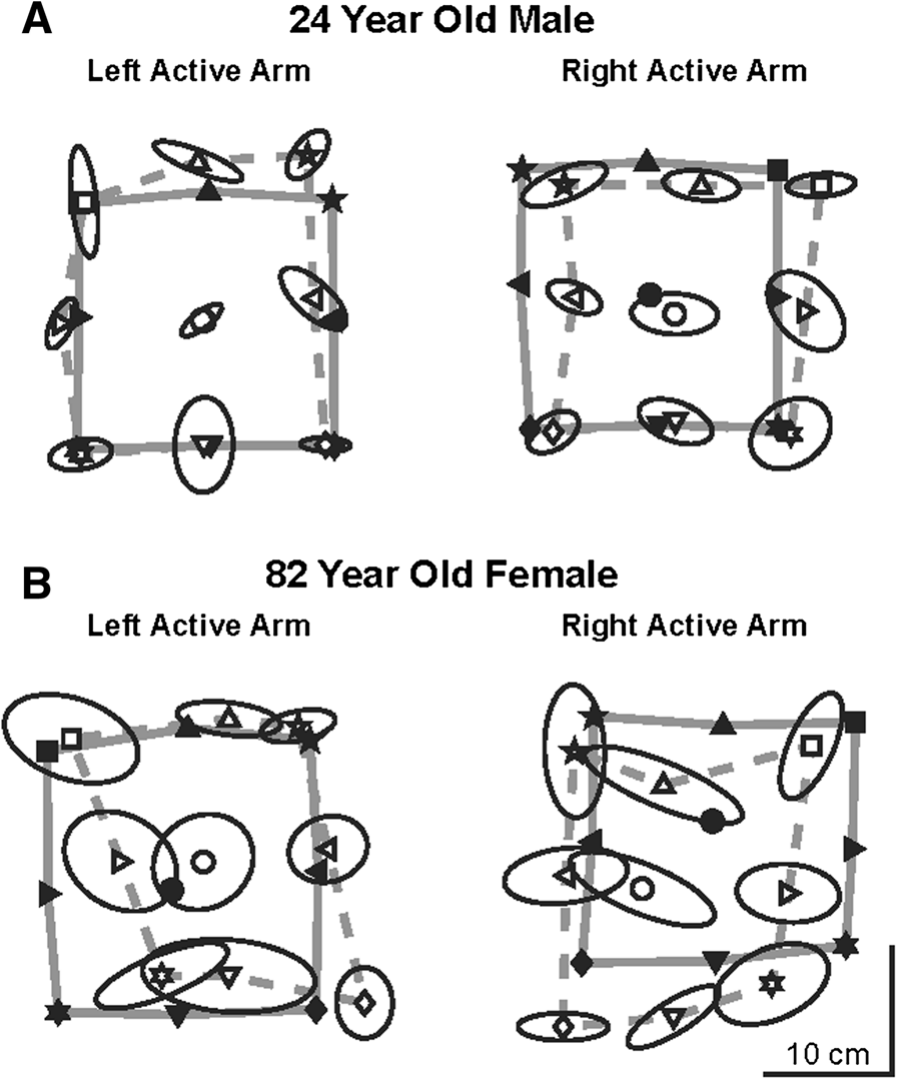
**Position matching behavior of two representative participants, a 24 year old male (A) and an 82 year old female (B).** Each plot shows the mean hand position of the active arm (open symbols) superimposed on the passive arm (closed symbols) for each of the nine target locations. Thin black ellipses show the variability of the mean hand position of the active arm for each target. The areas enclosed by the solid and dashed grey lines show the matching areas of passive and active arms, respectively.

Figure 3 illustrates the performance of two representative participants, a 24 year old male (A) and an 82 year old female (B), in the arm position matching task. The 24 year old male exhibited mean Var_xy_ scores of 3.3 cm and 3.7 cm with the left and right active hands, respectively. The 82 year old female exhibited mean Var_xy_ scores of 4.1 cm with both active hands. A comparison of the areas subtended by the active (dashed grey lines) and passive (solid grey lines) hands highlights the relative contraction or expansion (C/E) of the target set. The 24 year old male did not exhibit obvious contraction or expansion of the workspace with either hand (C/E = 0.98 and 0.97) whereas the 82 year old female displayed modest contraction of the workspace with both hands (C/E = 0.88 and 0.75). Both exemplar participants exhibited relatively small systematic shifts, with the 24 year old male exhibiting smaller shifts (Shift_xy_ = 3.7 and 2.0 cm) than the 82 year old female (Shift_xy_ = 5.1 and 3.7 cm). Finally, absolute errors displayed by each exemplar participant reflected their overall performance on the other three parameters. The 24 year old male obtained absolute *xy* errors of 4.4 cm and 3.5 cm as compared to the 82 year old female, who had absolute *xy* errors of 5.8 cm and 4.8 cm.

**Figure 4:**
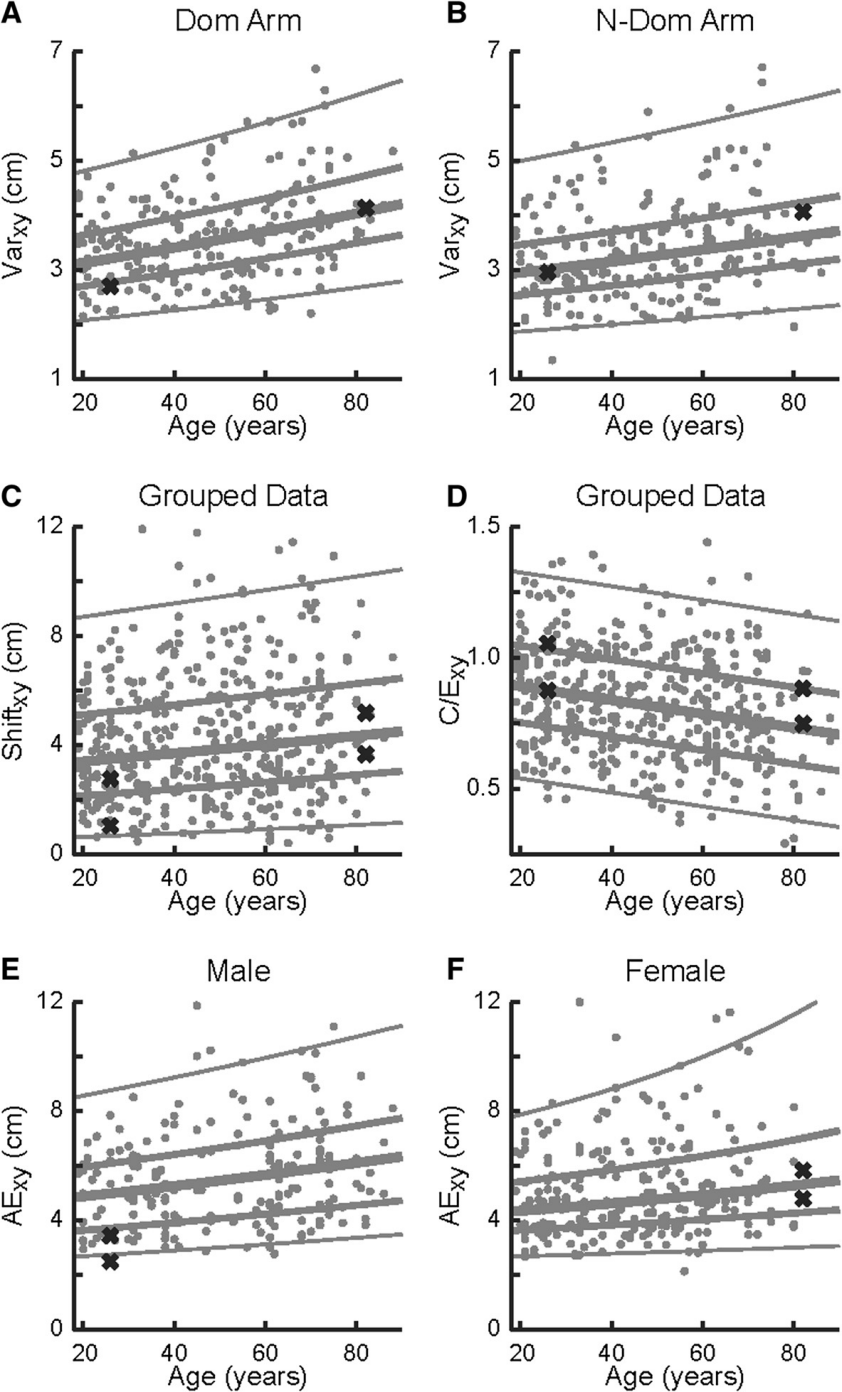
**Effect of age on hand-based parameters of position sense.** Each dot represents one arm of a participant. **A**, **B**, Variability (Var_*xy*_) of the dominant **(A)** and non-dominant **(B)** arms. **C**, Systematic shifts (Shift_xy_). **D**, Spatial Contraction/Expansion (C/E). **E**, **F**, Absolute error (AE_xy_) of males **(E)** and females **(F)**. In each panel, parameter values are plotted in their native untransformed units even if they were transformed to attain a normal distribution of residuals during the regression analyses. Values of the representative participants from Figure 3 are shown with an X. Lines show the median (thick line), inter-quartile range (medium thickness lines), and central 95% confidence interval (thin lines) obtained from the regression analysis.

Trial-to-trial variability in matching the position of the limb at a given target location was generally influenced by age (Var_x_, Var_y_, Var_xy_: *P* ≤ 0.001) and test-hand (Var_x_, Var_xy_: *P* ≤ 0.004) but not sex (Table 3). Log transforms were required for all variability regressions, denoting that both the mean and range of variability increased exponentially with age. The effects of age and test-hand on Var_xy_ is displayed in Figures 4A and B. Across adulthood (18 to 90 years of age), median performance in Var_xy_ increased from 3.1 cm to 4.2 cm (36%) when the test-hand was the dominant hand (*P* < 10^-5^) and from 2.9 cm to 3.7 cm (27%) when the test-hand was the non-dominant hand (*P* = 0.001). Similarly, when the test-hand was the dominant hand the range of Var_xy_ between the 5th and 95th percentiles increased from 2.5 cm at 18 years of age to 3.4 cm at 90 years of age (36%). When the test-hand was the non-dominant hand, the range increased from 2.4 cm to 3.0 cm (27%) between 18 and 90 years of age.

**Table 3:** Model fits and percentiles for hand-based parameters of position sense

**Param**	**Group**	**Trans**		**Model fit**		**Percentiles**								
			***P***	**Slope**	**Bias**	**1**	**2.5**	**5**	**25**	**50**	**75**	**95**	**97.5**	**99**
Var_x_	Dom	log	<10^-5^	0.0044	0.935	–0.566	–0.479	–0.406	–0.151	–0.006	0.169	0.418	0.458	0.489
Var_x_	N-Dom	log	0.001	0.0036	0.873	–0.729	–0.528	–0.462	–0.191	–0.005	0.178	0.456	0.529	0.652
Var_y_	All	log	<10^-4^	0.0030	0.268	–0.604	–0.472	–0.400	–0.175	–0.011	0.164	0.425	0.505	0.642
Var_xy_	Dom	log	<10^-5^	0.0042	1.056	–0.492	–0.410	–0.375	–0.146	–0.001	0.150	0.400	0.430	0.459
Var_xy_	N-Dom	log	0.001	0.0033	1.023	–0.616	–0.463	–0.432	–0.157	–0.007	0.152	0.414	0.519	0.595
Shift_x_	All	—	0.673	—	—	–9.184	–7.765	–6.501	–3.221	–0.550	2.534	7.242	8.098	9.425
Shift_y_	Dom	—	0.135	—	—	–5.716	–4.684	–4.172	–1.464	0.015	1.627	3.614	5.143	5.610
Shift_y_	N-Dom	—	0.009	–0.0241	–0.093	–4.973	–4.058	–3.641	–1.622	–0.034	1.567	3.979	4.928	6.699
Shift_xy_	All	sqrt	0.009	0.0040	1.784	–1.241	–1.066	–0.926	–0.399	–0.022	0.397	0.917	1.087	1.303
C/E_xy_	All	—	<10^-5^	–0.0026	0.954	–0.416	–0.362	–0.318	–0.148	–0.009	0.146	0.350	0.425	0.513
AE_xy_	Male	log	0.001	0.0038	1.479	–0.639	–0.569	–0.488	–0.265	0.030	0.229	0.483	0.593	0.661
AE_xy_	Female	inv	0.012	–0.0007	0.247	0.146	0.141	0.113	0.042	–0.003	–0.049	–0.097	–0.106	–0.119

*Abbreviations:* Var, variability; C/E, spatial contraction/expansion; AE, absolute error. For parameters with a significant effect of sex or active-hand, model fits and percentiles are given for each group. None of the parameters had a significant effect of both sex and active-hand.

Unlike variability, systematic shifts (Shift) in matching performance were not consistently affected by age (Table 3). Shifts along the x dimension (Shift_x_) were not significantly influenced by age, sex, or test-hand, whereas linear shifts (Shift_xy_) increased with age (*P* = 0.009) but were unaffected by sex or test-hand. Shift_y_ was influenced by the test-hand (*P* < 10^-5^) but not sex, thus the dominant and non-dominant hands were treated separately. Shift_y_ for the non-dominant hand was influenced by age (*P* = 0.009), whereas Shift_y_ for the dominant hand did not have a significant regression with age (*P* = 0.135). Figure 4C highlights the performance of all subjects for Shift_xy_. From 18 to 90 years of age, participants exhibited a significant increase from 3.4 cm to 4.5 cm (34%) in their median Shift_xy_. However, this influence of age is much smaller than the range in performance between 5th and 95th percentiles, which was 6.8 cm at 18 years of age and 7.9 cm at 90 years of age.

Contraction/expansion (C/E) value was the only hand-based parameter not influenced by sex or test-hand (Table 3). A significant regression was observed between C/E and age (*P* < 10^-5^) such that there was a 21% increase in contraction of the matching workspace across adulthood. Figure 4D shows the effect of age on C/E_xy_. At age 20, median performance was 0.89 meaning that the spatial area of the active hand was 89% of the spatial area of the passive hand moved by the robot. In contrast, median performance was 0.73 at age 80 years old. The influence of age on C/E was again much smaller than the difference between 5th to 95th percentile values, which was equal to 0.67 at a given age.

Absolute errors in the *xy* dimension (AE_xy_) exhibited a significant effect of sex (*P* = 0.003) but not test-hand, thus males and females were separated for the regression analyses (Table 3). Figure 4E illustrates that male participants displayed a significant increase from 4.8 cm to 6.3 cm (31%) in their median AE_xy_ across adulthood (*P* = 0.001). Similarly, Figure 4F shows that female participants exhibited a significant increase 3.8 cm to 4.6 cm (22%) in their median AE_xy_ across adulthood (*P* = 0.012). Again, the influence of age on AE was much smaller than the range of values for males and females at a given age.

### Joint-based parameter analysis

**Figure 5:**
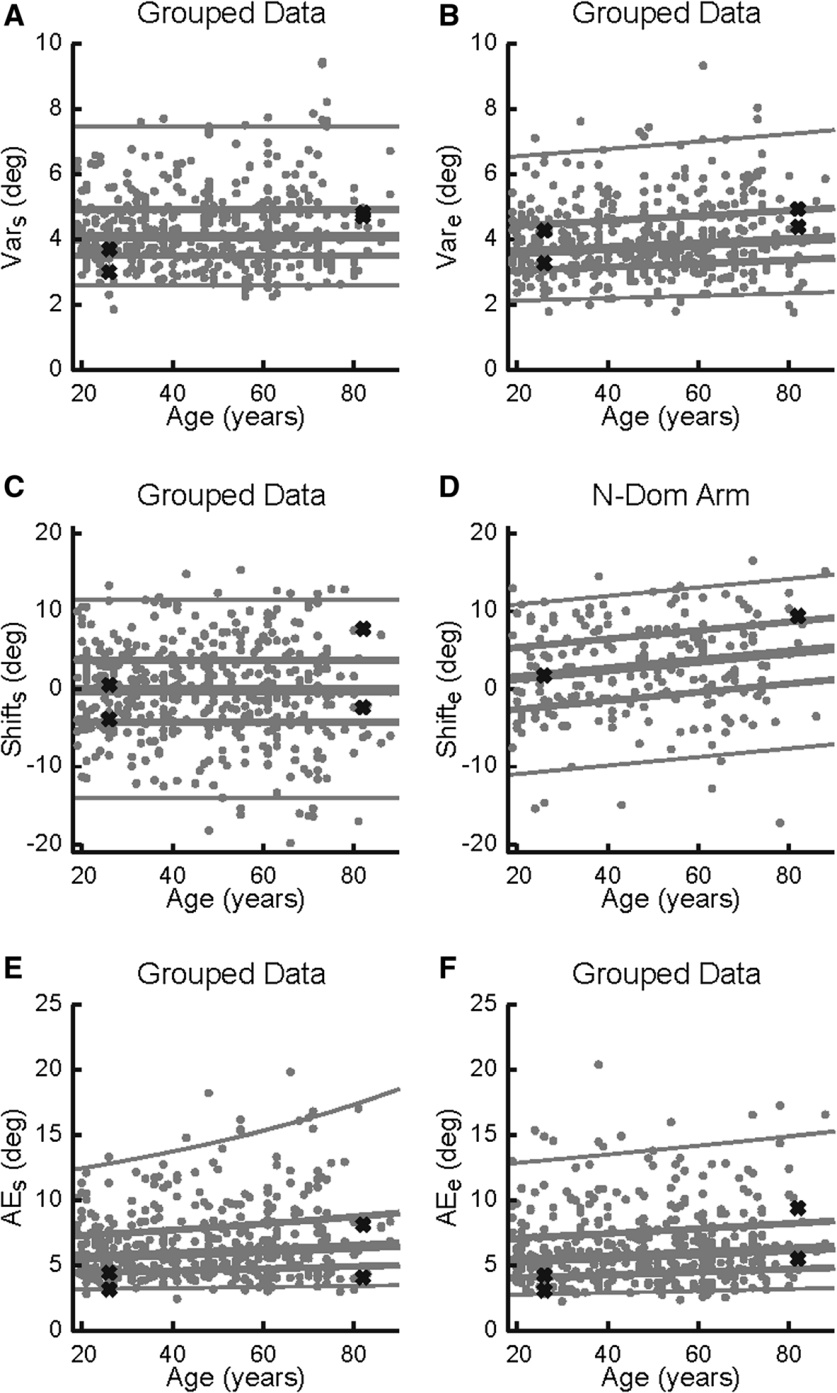
**Effects of age on joint-based parameters of position sense. A**, **B**, Variability observed at the shoulder **(A)** and elbow **(B)**. **C**, Systematic shifts at the shoulder (grouped data). *D*, Systematic shifts at the elbow non-dominant arm. **E***,***F***,* Absolute errors at the shoulder and elbow. Parameter values are plotted in their native, untransformed units. Values of the representative participants from Figure 3 are shown with Xs. Lines show the median (thick line), inter-quartile range (medium thickness lines), and central 95% confidence interval (thin lines) obtained from the regression analysis.

Figure 5 and Table 4 show the statistical influence of increasing age on joint-based parameters. We did not find a significant effect of sex or test-hand for variability at the shoulder and elbow. Furthermore, variability at the shoulder did not show a significant change with age (Figure 5A), whereas we observed a 0.5° (13%) increase in variability at the elbow across adulthood (*P* = 0.030; Figure 5B). Age-related changes in variability at the elbow were much smaller than inter-subject differences, which could be as small as 2° for some subjects and as large as 7° for other subjects.

**Table 4:** Model fits and percentiles for joint-based parameters of position sense

**Param**	**Group**	**Trans**	**Model fit**			**Percentiles**								
			***P***	**Slope**	**Bias**	**1**	**2.5**	**5**	**25**	**50**	**75**	**95**	**97.5**	**99**
Var_s_	All	—	0.725	—	—	2.317	2.605	2.821	3.507	4.075	4.917	6.593	7.474	7.783
Var_e_		log	0.030	0.0017	1.265	–0.656	–0.543	–0.442	–0.184	–0.017	0.188	0.488	0.582	0.658
Shift_s_		—	0.102	—	—	–16.33	–14.05	–11.42	–4.293	–0.123	3.731	10.38	11.47	12.81
Shift_e_	Dom	—	0.064	—	—	–15.15	–13.03	–10.15	–4.540	–0.439	3.293	8.992	10.29	11.81
Shift_e_	N-Dom	—	0.015	0.0549	–0.064	–16.73	–12.14	–8.709	–3.785	0.265	4.192	8.595	9.694	11.12
AE_s_		inv	0.024	–0.0004	0.190	0.161	0.132	0.100	0.044	–0.004	–0.045	–0.091	–0.102	–0.105
AE_e_		log	0.028	0.0024	1.650	–0.809	–0.687	–0.628	–0.296	–0.018	0.260	0.711	0.856	0.982

*Abbreviations:* Var, variability; AE, absolute error. For parameters with a significant effect of sex or active-hand, model fits and percentiles are given for each group. None of the parameters had a significant effect of both sex and active-hand.

Systematic shifts at the shoulder did not exhibit a significant effect of sex or test-hand, nor did they change significantly with age (Figure 5C). Systematic shifts at the elbow, however, displayed a significant effect of test-hand (*P* < 10^-5^). Although systematic shifts at the elbow when the test-hand was the dominant hand did not exhibit a significant effect of aging (data not shown), systematic shifts at the elbow for the non-dominant hand increased by 4.0° across adulthood (*P* = 0.015; Figure 5D). The range of shifts at any given age (approximately 20 degrees) was much larger than the change that occurred with increases in age across adulthood.

Absolute errors at the shoulder and elbow did not display significant effects of sex or test-hand. Regression analyses showed a significant increase of 1.0° (18%) across adulthood for median absolute errors at the shoulder (*P* = 0.024; Figure 5E). Regression analysis of absolute errors at the elbow showed a significant increase of 1.0° (19%) across adulthood (*P* = 0.028; Figure 5F). Changes in absolute error at both the shoulder and elbow were much smaller than the inter-subject variability at any age.

## Discussion

The present study quantified position sense of the upper arm in a cohort of subjects spanning many decades of life. Our findings demonstrate that position sense tends to diminish with increasing age across adulthood. The vast majority of the parameters changed with age (hand-based: Var_x_, Var_y_, Var_xy_, Shift_y_, Shift_xy_, C/E_xy_, AE_x_; joint-based: Var_e_, Shift_e_, AE_s_, AE_e_), with the largest relative effect for the hand-based parameter Var_xy_ (36%). Several parameters also exhibited effects of sex (hand-based: AE_xy_) and test-hand (hand-based: Var_x_, Var_xy_, Shift_y_, Var_x_; joint-based: Shift_e_).

The effect of age on position sense is consistent with most previous reports in the literature [20-23], though it is not clear why the large cohort of subjects in a study of position sense of the metacarpophalangeal joint did not show an effect of age [24]. Theoretically, aging should have similar influences on sensory receptors (muscle spindles and cutaneous receptors) and cortical grey matter mediating position sense at all joints. This would make it unlikely that age would influence position sense for proximal and not distal joints of the upper limb. The conflicting results may reflect different sensitivities of the assessments used in the two studies. Alternatively, aging may cause smaller declines in sensitivity of cutaneous receptors, resulting in smaller age-related declines in distal joints, which have higher densities of cutaneous receptors.

Over the years, position sense of the upper extremity has been measured many different ways. Some studies assess position sense at individual joints, by identifying the smallest noticeable movement of the joint [42,55,56], replicating a specific joint angle [38,57-60] or matching the joint angle in the other limb [22,23,38,59,60]. In general, position sense tends to be better for the more proximal joints as compared to distal ones [61], reflecting differences in the number of muscle spindles spanning each joint [62]. Other studies have examined whole-limb position sense by quantifying the ability of subjects to estimate the position of the hand [20,63]. As no muscle spans the shoulder, elbow and wrist, subjects must integrate afferent signals from several muscles to estimate hand position and this estimate is influenced by limb geometry [62].

Differences seen across studies on the influence of aging on position sense may reflect variations in experimental approach. Some studies measured single joint angle errors [21-23], whereas one measured the location of the hand [20]. A further potential confounding factor is very large inter-subject variability in position sense. For example, Adamo et al., [22] demonstrated that elderly subjects concurrently matching elbow angle had an average error of 3.04 degrees with a standard deviation of 1.38 degrees. We also found considerable inter-subject variability in task performance making it more difficult to statistically identify whether age or other factors influence position sense.

Another difference across studies on aging is whether assessment of position sense was made at a single joint versus whole limb. The latter involves greater complexity because position sense is estimated by integrating sensory signals from many muscles spanning different joints with internal knowledge of segment lengths. We chose to instruct subjects to match their whole-limb rather than joint position because we are behaviourally more concerned about the position of the limb or hand than the particular angle of an individual joint. These differences in the complexity of the task design (multi-joint versus single-joint) and behavioural goals (matching hand position versus joint angle) may contribute to why we found that many parameters were sensitive to increases in age across adulthood.

Our experimental apparatus and study design allowed examination of both hand- and joint-based features. Overall, the majority of our hand- and joint-based parameters studied demonstrated declines in position sense with age. However, analysis of the shoulder, in particular, revealed results that appear somewhat discrepant. Despite the fact that the AE at the shoulder worsens with age, the other shoulder-based parameters (Var, Shift) did not significantly change with age. AE is a measurement that has been used by many other authors when evaluating position sense [20,22,23]. If one considers how the parameters AE, Var and Shift are calculated, we conclude that one should consider AE as a more global measure of position sense because AE is sensitive to both trial-to-trial variability and systematic shifts. Thus, the effects of age reached significance for AE but not these other parameters.

Differences in motor skills of the dominant and non-dominant hand are quite apparent in many activities such as throwing or catching a ball. Although the dominant limb is often better in motor tasks, in some cases the non-dominant limb displays better performance such as end-point accuracy during reaching [63,64]. We found a significant effect of test-hand in three of the hand-based parameters (Var_x_, Var_xy_, Shift_xy_). In each case, performance was better when the robot moved the dominant hand and subjects matched with their non-dominant hand, which is consistent with previous studies [38,60]. As well, age had less of an influence on subject performance when the non-dominant hand was used to match the position of the dominant hand. We found Shift_elb_ was slightly worse when the non-dominant limb was the active arm, although this may reflect the task instruction focused on position sense of the whole-limb and not the joints. Overall, however, position sense appears to be better when relying on information passing from the dominant to the non-dominant limb. However, the current study was not specifically designed to address the neural mechanisms linking hemispheric dominance to position sense. Future studies that are specifically designed to address this issue would be valuable to help better understand hemispheric differences in the processing of proprioceptive information.

With respect to sex, only the hand-based parameter AE_xy_ demonstrated a significant difference between males and females. As described for measures of shoulder performance, AE captures both variability and systematic shifts, thus it may be more sensitive for identifying group differences. Another group has recently reported sex differences in a position sense task, although this occurred only after subjects followed a protocol aimed at significantly fatiguing muscles in the upper extremity [65]. Given the relationship between sex and body size, measures such as height, weight, and body mass index may have exhibited additional relationships with robotic parameters. However, a detailed analysis of these additional factors was beyond the scope of the current study.

Although we found age, sex and test-hand influenced position sense, it is clear that these factors only explain a small proportion of inter-subject variability. For example, changes in position sense across adulthood tended to be only 10 to 30% of the range observed for the 5th to 95th percentile performance at a given age. Inter-rater reliability of the hand-based parameters was generally good although that analysis included both healthy controls and subjects with stroke [26]. Further work is required to identify how much of the inter-subject variability reflects actual differences in position sense across subjects and whether such differences impact or correlate with sensorimotor performance such as throwing accuracy or fine motor skills.

## Conclusion

The present study identified how age, sex and test-hand impacts position sense and provides new knowledge on this sensory process. Most hand- and joint-based parameters examined in this study indicated that subject performance generally declined with increasing age across adulthood. There also appears to be effects of test-hand and sex (to a lesser extent) on some attributes of position sense. Such information provides a basis for understanding impairments in position sense due to neurological disorders. Our previous research identified whether individual subjects with stroke had deficits in position sense [26,27]. The present regression models will improve these analyses, creating patient-specific estimates of healthy performance based on age, sex and test-hand.

## Competing interests

SHS is the Co-founder and Chief Scientific Officer of BKIN Technologies that commercializes the robotic technology used in this study.

## Authors’ contributions

TMH helped conceive of the concept for the manuscript, performed data analysis and participated in the writing of the manuscript. SHS assisted with data analysis and participated in writing of the manuscript. SPD helped conceive of the concept for the manuscript, assisted with data collection and participated in the writing of the manuscript. All authors approved the final version of the manuscript.

## References

[B1] Sherrington CS On the proprio-ceptive system, especially in its reflex aspect Brain 1907 29 467 482 10.1093/brain/29.4.467

[B2] Proske U Gandevia SC The kinaesthetic senses J Physiol 2009 587 4139 4146 10.1113/jphysiol.2009.175372 19581378 PMC2754351

[B3] Proske U Kinesthesia: the role of muscle receptors Muscle Nerve 2006 34 545 558 10.1002/mus.20627 16897766

[B4] Proske U What is the role of muscle receptors in proprioception? Muscle Nerve 2005 31 780 787 10.1002/mus.20330 15818635

[B5] Matthews PB Where does Sherrington's "muscular sense" originate? Muscles, joints, corollary discharges? Annu Rev Neurosci 1982 5 189 218 10.1146/annurev.ne.05.030182.001201 6462096

[B6] McCloskey DI Kinesthetic Sensibility Physiol Rev 1978 58 763 820 360251 10.1152/physrev.1978.58.4.763

[B7] Burke JR Schutten MC Koceja DM Kamen G Age-dependent effects of muscle vibration and the Jendrassik maneuver on the patellar tendon reflex response Arch Phys Med Rehabil 1996 77 600 604 10.1016/S0003-9993(96)90302-0 8831479

[B8] Kim GH Suzuki S Kanda K Age-related physiological and morphological changes of muscle spindles in rats J Physiol 2007 582 525 538 10.1113/jphysiol.2007.130120 17495047 PMC2075321

[B9] Miwa T Miwa Y Kanda K Dynamic and static sensitivities of muscle spindle primary endings in aged rats to ramp stretch Neurosci Lett 1995 201 179 182 10.1016/0304-3940(95)12165-X 8848247

[B10] Liu JX Eriksson PO Thornell LE Pedrosa-Domellof F Fiber content and myosin heavy chain composition of muscle spindles in aged human biceps brachii J Histochem Cytochem 2005 53 445 454 10.1369/jhc.4A6257.2005 15805419

[B11] Swash M Fox KP The effect of age on human skeletal muscle. Studies of the morphology and innervation of muscle spindles J Neurol Sci 1972 16 417 432 10.1016/0022-510X(72)90048-2 4261815

[B12] Kararizou E Manta P Kalfakis N Vassilopoulos D Morphometric study of the human muscle spindle Anal Quant Cytol Histol 2005 27 1 4 15794446

[B13] Aydog ST Korkusuz P Doral MN Tetik O Demirel HA Decrease in the numbers of mechanoreceptors in rabbit ACL: the effects of ageing Knee Surg Sports Traumatol Arthrosc 2006 14 325 329 10.1007/s00167-005-0673-2 16133439

[B14] Morisawa Y Morphological study of mechanoreceptors on the coracoacromial ligament J Orthop Sci 1998 3 102 110 10.1007/s007760050029 9654563

[B15] Good CD Johnsrude IS Ashburner J Henson RN Friston KJ Frackowiak RS A voxel-based morphometric study of ageing in 465 normal adult human brains Neuroimage 2001 14 21 36 10.1006/nimg.2001.0786 11525331

[B16] Quiton RL Roys SR Zhuo J Keaser ML Gullapalli RP Greenspan JD Age-related changes in nociceptive processing in the human brain Ann N Y Acad Sci 2007 1097 175 178 10.1196/annals.1379.024 17413021

[B17] Goble DJ Coxon JP Van Impe A Geurts M Van Hecke W Sunaert S Wenderoth N Swinnen SP The neural basis of central proprioceptive processing in older versus younger adults: an important sensory role for right putamen Hum Brain Mapp 2012 33 895 908 10.1002/hbm.21257 21432946 PMC6870471

[B18] Goble DJ Mousigian MA Brown SH Compromised encoding of proprioceptively determined joint angles in older adults: the role of working memory and attentional load Exp Brain Res 2012 216 35 40 10.1007/s00221-011-2904-8 22006273

[B19] Goble DJ Coxon JP Wenderoth N Van Impe A Swinnen SP Proprioceptive sensibility in the elderly: degeneration, functional consequences and plastic-adaptive processes Neurosci Biobehav Rev 2009 33 271 278 10.1016/j.neubiorev.2008.08.012 18793668

[B20] Stelmach GE Sirica A Aging and Proprioception Age 1986 9 99 103 10.1007/BF02432281

[B21] Ferrell WR Crighton A Sturrock RD Age-dependent changes in position sense in human proximal interphalangeal joints Neuroreport 1992 3 259 261 10.1097/00001756-199203000-00011 1515581

[B22] Adamo DE Martin BJ Brown SH Age-related differences in upper limb proprioceptive acuity Percept Mot Skills 2007 104 1297 1309 17879664 10.2466/pms.104.4.1297-1309

[B23] Adamo DE Alexander NB Brown SH The influence of age and physical activity on upper limb proprioceptive ability J Aging Phys Act 2009 17 272 293 19799100 10.1123/japa.17.3.272

[B24] Kokmen E Bossemeyer RW Williams WJ Quantitative Evaluation of Joint Motion Sensation in an Aging Population J Gerontol 1978 33 62 67 10.1093/geronj/33.1.62 618968

[B25] Debert CT Herter TM Scott SH Dukelow S Robotic assessment of sensorimotor deficits after traumatic brain injury J Neurol Phys Ther 2012 36 58 67 10.1097/NPT.0b013e318254bd4f 22592061

[B26] Dukelow SP Herter TM Moore KD Demers MJ Glasgow JI Bagg SD Norman KE Scott SH Quantitative assessment of limb position sense following stroke Neurorehabil Neural Repair 2010 24 178 187 10.1177/1545968309345267 19794134

[B27] Dukelow SP Herter TM Bagg SD Scott SH The independence of deficits in position sense and visually guided reaching following stroke J Neuroeng Rehabil 2012 9 72 10.1186/1743-0003-9-72 23035968 PMC3543214

[B28] de Weerdt W Lincoln NB Harrison MA Prediction of arm and hand function recovery in stroke patients Int J Rehabil Res 1987 10 110 112 3503832

[B29] Kuffosky A Wadell I Nilsson BY The relationship between sensory impairment and motor recovery in patients with hemiplegia Scand J Rehabil Med 1982 14 27 32 7063817

[B30] La Joie WJ Reddy NM Melvin JL Somatosensory evoked potentials: their predictive value in right hemiplegia Arch Phys Med Rehabil 1982 63 223 226 7073461

[B31] Pavot AP Ignacio DR Kuntavanish A Lightfoote WE 2nd The prognostic value of somatosensory evoked potentials in cerebrovascular accidents Electromyogr Clin Neurophysiol 1986 26 333 340 3780520

[B32] Wade DT Langton-Hewer R Wood VA Skilbeck CE Ismail HM The hemiplegic arm after stroke: measurement and recovery J Neurol Neurosurg Psychiatry 1983 46 521 524 10.1136/jnnp.46.6.521 6875585 PMC1027442

[B33] Cowell PE Turetsky BI Gur RC Grossman RI Shtasel DL Gur RE Sex differences in aging of the human frontal and temporal lobes J Neurosci 1994 14 4748 4755 8046448 10.1523/JNEUROSCI.14-08-04748.1994 PMC6577197

[B34] Coffey CE Lucke JF Saxton JA Ratcliff G Unitas LJ Billig B Bryan RN Sex differences in brain aging: a quantitative magnetic resonance imaging study Arch Neurol 1998 55 169 179 10.1001/archneur.55.2.169 9482358

[B35] Xu J Kobayashi S Yamaguchi S Iijima K Okada K Yamashita K Gender effects on age-related changes in brain structure AJNR Am J Neuroradiology 2000 21 112 118 PMC7976349 10669234

[B36] Sainburg RL Evidence for a dynamic-dominance hypothesis of handedness Exp Brain Res 2002 142 241 258 10.1007/s00221-001-0913-8 11807578 PMC10710695

[B37] Roy EA MacKenzie C Handedness effects in kinesthetic spatial location judgements Cortex 1978 14 250 258 10.1016/S0010-9452(78)80051-3 679706

[B38] Goble DJ Brown SH Upper limb asymmetries in the matching of proprioceptive versus visual targets J Neurophysiol 2008 99 3063 3074 10.1152/jn.90259.2008 18436632

[B39] Goble DJ Noble BC Brown SH Proprioceptive target matching asymmetries in left-handed individuals Exp Brain Res 2009 197 403 408 10.1007/s00221-009-1922-2 19572124

[B40] Goble DJ Brown SH Upper limb asymmetries in the perception of proprioceptively determined dynamic position sense J Exp Psychol Hum Percept Perform 2010 36 768 775 20515203 10.1037/a0018392

[B41] Jones SA Cressman EK Henriques DY Proprioceptive localization of the left and right hands Exp Brain Res 2010 204 373 383 10.1007/s00221-009-2079-8 19921158

[B42] Bickley LS Szilagyi PG Bates' Guide to Physical Examination and History Taking (9th Edition) 2007 Hagerstown, MD Lippincott: Williams and Wilkins

[B43] Tiffen J Asher EJ The purdue pegboard: norms and studies of reliability and validity J Appl Psychol 1948 32 234 247 18867059 10.1037/h0061266

[B44] Oldfield RC The assessment and analysis of handedness: the Edinburgh inventory Neuropsychologia 1971 9 97 113 10.1016/0028-3932(71)90067-4 5146491

[B45] Szaflarski JP Binder JR Possing ET McKiernan KA Ward BD Hammeke TA Language lateralization in left-handed and ambidextrous people: fMRI data Neurology 2002 59 238 244 10.1212/WNL.59.2.238 12136064

[B46] Coderre AM Zeid AA Dukelow SP Demmer MJ Moore KD Demers MJ Bretzke H Herter TM Glasgow JI Norman KE Bagg SD Scott SH Assessment of upper-limb sensorimotor function of subacute stroke patients using visually guided reaching Neurorehabilitation and neural repair 2010 24 528 541 10.1177/1545968309356091 20233965

[B47] Scott SH Apparatus for measuring and perturbing shoulder and elbow joint positions and torques during reaching J Neurosci Methods 1999 89 119 127 10.1016/S0165-0270(99)00053-9 10491942

[B48] Semrau JA Herter TM Scott SH Dukelow SP Robotic identification of kinesthetic deficits after stroke Stroke 2013 44 3414 3421 10.1161/STROKEAHA.113.002058 24193800

[B49] Carpenter JE Blasier RB Pellizzon GG The effects of muscle fatigue on shoulder joint position sense Am J Sports Med 1998 26 262 265 9548121 10.1177/03635465980260021701

[B50] Aydin T Yildiz Y Yanmis I Yildiz C Kalyon TA Shoulder proprioception: a comparison between the shoulder joint in healthy and surgically repaired shoulders Arch Orthop Trauma Surg 2001 121 422 425 10.1007/s004020000245 11510910

[B51] Dover G Powers ME Cryotherapy does not impair shoulder joint position sense Arch Phys Med Rehabil 2004 85 1241 1246 10.1016/j.apmr.2003.11.030 15295747

[B52] Lonn J Crenshaw AG Djupsjobacka M Pedersen J Johansson H Position sense testing: influence of starting position and type of displacement Arch Phys Med Rehabil 2000 81 592 597 10807097 10.1016/s0003-9993(00)90040-6

[B53] Scott SH Dukelow SP Potential of robots as next-generation technology for clinical assessment of neurological disorders and upper-limb therapy J Rehabilitation Res Design 2011 48 335 354 10.1682/JRRD.2010.04.0057 21674387

[B54] Lonn J Crenshaw AG Djupsjobacka M Johansson H Reliability of position sense testing assessed with a fully automated system Clin Physiol 2000 20 30 37 10.1046/j.1365-2281.2000.00218.x 10651789

[B55] Refshauge KM Collins DF Gandevia SC The detection of human finger movement is not facilitated by input from receptors in adjacent digits J Physiol 2003 551 371 377 10.1113/jphysiol.2003.045997 12815183 PMC2343141

[B56] Collins DF Refshauge KM Todd G Gandevia SC Cutaneous receptors contribute to kinesthesia at the index finger, elbow, and knee J Neurophysiol 2005 94 1699 1706 10.1152/jn.00191.2005 15917323

[B57] Fuentes CT Bastian AJ Where is your arm? Variations in proprioception across space and tasks J Neurophysiol 2010 103 164 171 10.1152/jn.00494.2009 19864441 PMC4116392

[B58] Goble DJ Noble BC Brown SH Where was my arm again? Memory-based matching of proprioceptive targets is enhanced by increased target presentation time Neurosci Lett 2010 481 54 58 10.1016/j.neulet.2010.06.053 20600603

[B59] Walsh LD Hesse CW Morgan DL Proske U Human forearm position sense after fatigue of elbow flexor muscles J Physiol 2004 558 705 715 10.1113/jphysiol.2004.062703 15181165 PMC1664958

[B60] Goble DJ Lewis CA Brown SH Upper limb asymmetries in the utilization of proprioceptive feedback Exp Brain Res 2006 168 307 311 10.1007/s00221-005-0280-y 16311728

[B61] Hall LA McCloskey DI Detections of movements imposed on finger, elbow and shoulder joints J Physiol 1983 335 519 533 6875893 10.1113/jphysiol.1983.sp014548 PMC1197367

[B62] Scott SH Loeb GE The computation of position sense from spindles in mono- and multiarticular muscles J Neurosci 1994 14 7529 7540 7996193 10.1523/JNEUROSCI.14-12-07529.1994 PMC6576884

[B63] Bagesteiro LB Sainburg RL Nondominant arm advantages in load compensation during rapid elbow joint movements J Neurophysiol 2003 90 1503 1513 10.1152/jn.00189.2003 12736237 PMC10704424

[B64] Wang J Sainburg RL The dominant and nondominant arms are specialized for stabilizing different features of task performance Exp Brain Res 2007 178 565 570 10.1007/s00221-007-0936-x 17380323 PMC10702172

[B65] Emery K Cote JN Repetitive arm motion-induced fatigue affects shoulder but not endpoint position sense Exp Brain Res 2012 216 553 564 10.1007/s00221-011-2959-6 22124803

